# Bilateral sternoclavicular joint septic arthritis secondary to indwelling central venous catheter: a case report

**DOI:** 10.1186/1752-1947-2-131

**Published:** 2008-04-29

**Authors:** Charita Pradhan, Nicholas FS Watson, Nitin Jagasia, Ray Chari, Jane E Patterson

**Affiliations:** 1 Department of General Surgery, Kings Mill Hospital, Mansfield, UK; 2 Department of Orthopaedic Surgery, Kings Mill Hospital, Mansfield, UK

## Abstract

**Introduction:**

Septic arthritis of the sternoclavicular joint is rare, comprising approximately 0.5% to 1% of all joint infections. Predisposing causes include immunocompromising diseases such as diabetes, HIV infection, renal failure and intravenous drug abuse.

**Case presentation:**

We report a rare case of bilateral sternoclavicular joint septic arthritis in an elderly patient secondary to an indwelling right subclavian vein catheter. The insidious nature of the presentation is highlighted. We also review the literature regarding the epidemiology, investigation and methods of treatment of the condition.

**Conclusion:**

SCJ infections are rare, and require a high degree of clinical suspicion. Vague symptoms of neck and shoulder pain may cloud the initial diagnosis, as was the case in our patient. Surgical intervention is often required; however, our patient avoided major intervention and settled with parenteral antibiotics and washout of the joint.

## Introduction

Septic arthritis of the sternoclavicular joint (SCJ) is rare, comprising approximately 0.5% to 1% of all joint infections [1]. Infection tends to develop insidiously, and is frequently complicated by osteomyelitis. Predisposing causes include immunocompromising diseases such as diabetes, human immunodeficiency virus (HIV) infection, renal failure and intravenous drug abuse. One of the rarer causes is catheterisation of the subclavian vein.

## Case presentation

An 85-year-old man was admitted with a 3-week history of projectile vomiting, decreased appetite and halitosis. He was otherwise independent and had no medical comorbidities. On examination, he was observed to be cachectic and malnourished, with moderate renal impairment: urea 15.8 mg/dl; creatinine 197 μmol/l; white blood cell count (WBC) 9.1 × 10^9^/litre. A gastroscopy was performed, which diagnosed gastric outlet obstruction. Subsequent imaging by barium meal and computed tomography (CT) demonstrated a stricture in the second part of the duodenum due to a large fixed tumour, which was not considered amenable to curative surgical resection. As a result, the patient was scheduled for a palliative gastrojejunostomy.

During the surgical procedure, a multilumen right subclavian central venous catheter (CVC; Vygon, Cirencester, UK) was inserted by the anaesthetist with full aseptic precautions, using the Seldinger technique and under ultrasound guidance. No apparent difficulty in insertion or periprocedural complication was noted. Following confirmation of a satisfactory line position on plain chest radiography, the CVC was utilised for intravenous fluid infusion and supplemental postoperative parenteral nutrition.

The initial postoperative course was uneventful; however, on postoperative day 10, cellulitis was noticed around the insertion site of the catheter, which was still in situ. Accordingly, the CVC was removed and the tip sent for microbiological examination. The following day, the patient complained of severe pain in the left shoulder, with global painful limitation of left arm movements. Plain radiographs of the left shoulder joint were performed, demonstrating moderate osteoarthritic changes only.

Over postoperative days 12 and 13, the patient developed diarrhoea with intermittent pyrexia and raised inflammatory markers (erythrocyte sedimentation rate 94 mm/hour, C-reactive protein 112 mg/l, WBC 21.3 × 10^9^/litre). At this stage the cellulitis around the site of the previous CVC was noted to extend anteriorly over the sternum, and both peripheral blood cultures and the cultured CVC tip grew methicillin-resistant *Staphylococcus aureus *(MRSA), which was sensitive to vancomycin, gentamicin, rifampicin and tetracyclines, prompting treatment with intravenous vancomycin. A duplex ultrasound of the upper limb and neck veins was performed in order to exclude a subclavian venous thrombosis; however, an inflammatory mass was visualised over the sternum, with no apparent focal collection.

By day 15, pus was noted to be discharging from the CVC site, prompting a CT scan of the neck. This demonstrated a large inflammatory mass in the root of the neck extending into the superior mediastinum, with no evidence of abscess formation or any evidence of SCJ joint arthritis, either clinically or radiologically.

The patient received 2 weeks treatment with intravenous vancomycin (1 g daily) and oral rifampicin (600 mg daily) in accordance with local microbiology protocol. Regular monitoring of peak and trough serum vancomycin levels was performed to ensure therapeutic dosing. At the end of this period, the cellulitis had resolved and inflammatory markers were reduced; therefore, the patient was discharged.

**Figure 1:**
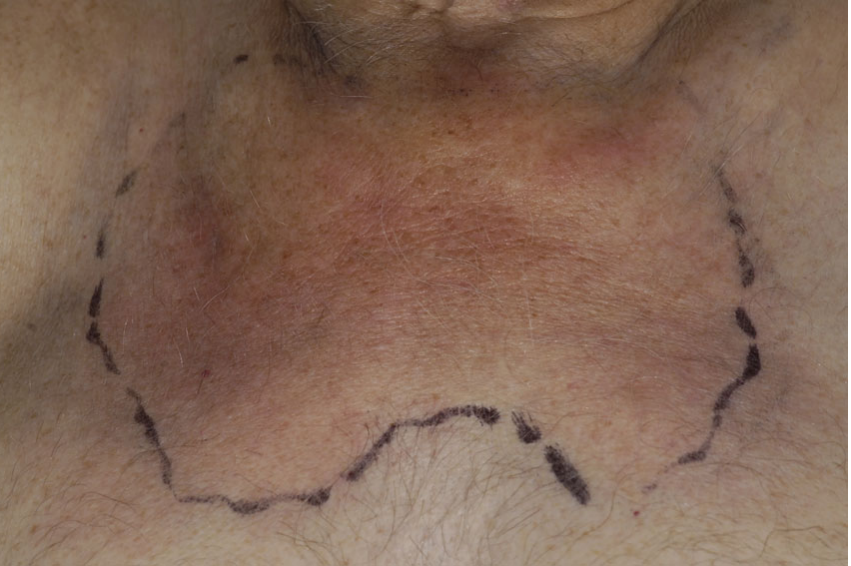
Clinical appearance of cellulitis overlying both sternoclavicular joints in association with septic arthritis.

**Figure 2:**
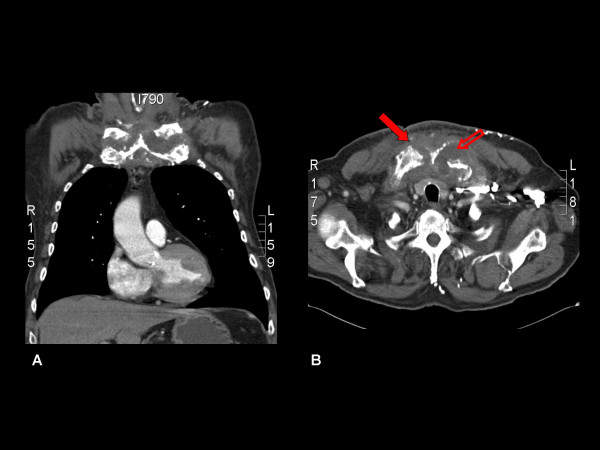
**Computed tomography scans demonstrating the destruction of the sternoclavicular joints bilaterally (solid arrow), with a large effusion of the left sternoclavicular joint (hollow arrow)**. (a) Coronal image; (b) sagittal image.

Once home, the patient continued to experience severe neck and shoulder pains, prompting his general practitioner to refer him again to the hospital approximately 1 month later. Clinical examination revealed a recurrence of the inflammatory mass over the sternum (Figure 1). The patient also complained of ongoing excruciating neck and shoulder pain requiring high doses of parenteral opiates, and inflammatory markers were markedly elevated. A further CT scan was performed, which demonstrated bilateral destruction of the SCJs and a large effusion of the left SCJ consistent with septic arthritis (Figure 2).

Thereafter, the patient was transferred to the care of the orthopaedic surgeons, who, after discussion with the local thoracic surgical unit, performed open surgical debridement of the left SCJ and obtained samples for microbiological and histological analyses. These showed osteolytic bone consistent with a diagnosis of suppurative osteomyelitis, and microbiology samples revealed a heavy growth of MRSA consistent with the previous CVC sepsis (antibiotic sensitivities as described previously). The patient was treated in hospital for a further 4 weeks, with combined intravenous vancomycin and oral rifampicin, leading to complete clinical resolution of his symptoms, and he was discharged with a further 4 weeks of single-agent oral rifampicin. No subsequent treatment has been required.

## Discussion

The SCJ is a synovial lined joint composed of the inferior medial clavicular head, the superior lateral notch of the manubrium and cartilage of the first rib. Clinical infections of this joint are exceedingly rare (<1% of all joint infections), but when present, result in abscess formation in 20% of cases [2,3]. The joint capsule is unable to distend so infection quickly spreads beyond the joint leading to fistula formation, cutaneous abscess or, rarely, mediastinitis [4,5]. Given the proximity to the mediastinum, pleural cavity and brachiocephalic structures, close liaison with thoracic surgeons is often required.

Infection of the SCJ mainly occurs in patients with immunocompromising conditions such as diabetes or chronic renal failure, in intravenous drug abusers and in those taking long-term steroids [1,6]. Infection in our patient occurred secondary to an indwelling subclavian catheter, in a patient predisposed to infection by a poor nutritional state and immunocompromised owing to his duodenal tumour. Venous catheter-related infections may be a result of haematogenous bacteraemia, but are more commonly held to result from direct inoculation of the joint during attempts at percutaneous vein catheterisation [7]. Others have suggested that colonisation of the catheter tract results in seeding of the SCJ capsule, which may have been traumatised at the time of insertion. The most commonly found organism is *Staphylococcus aureus*, which is demonstrated in up to half of all cases [8,9].

It is recognised that complications of *S. aureus *bacteraemia may be difficult to identify at the time of initial positive blood culture result, as was the case with our patient. To this end, Fowler et al. [10] have identified four clinical characteristics which may aid in the prediction of complicated infection. These are community acquisition, skin examination findings suggesting acute systemic infection, persistent fever at 72 hours and positive follow-up blood culture results at 48 to 96 hours. Of these, the presence of a positive follow-up blood culture was found to be the most important [10]. Follow-up blood cultures were not performed in our patient during initial admission and treatment. However, it is recommended that in patients with persisting *S. aureus *bacteraemia, the duration of antibiotic therapy should be prolonged.

Antibiotic selection and management of these patients should be guided by the local microbiology department, which can monitor local patterns of antibiotic susceptibility and resistance, determine the exact minimum inhibitory concentration of the chosen antibiotic, and monitor serum antibiotic levels in order to ensure therapeutic dosing.

In addition to vancomycin, a number of newer antimicrobial agents have demonstrated efficacy against MRSA [11]. These include the oxazolidinone, linezolid, which has both intravenous and oral formulations. In a recent animal study, linezolid was shown to be effective against experimentally induced MRSA mediastinitis, with no additional benefit from the addition of rifampicin [12]. Alternatives for the treatment of MRSA soft-tissue infection include daptomycin and the minocycline derivative, tigecycline.

The earliest presenting symptom of SCJ infection is neck and anterior chest pain followed by pyrexia, swelling and erythema over the neck and upper sternum. Often progression is insidious and may become apparent with shoulder discomfort (referred pain), as was the case with our patient. Conversely, it may present acutely with fever and septic shock owing to a high incidence of systemic bacteraemia associated with this condition. Superior vena caval obstruction due to mediastinitis may complicate the clinical picture.

In the late stages of SCJ infections, abscess formation with direct extension into the mediastinum and adjacent chest wall may occur in as many as 21% of cases [3,6]. The time interval between SCJ infection and symptoms vary and, therefore, a delay in diagnosis is not uncommon [1,6]. If the clinical diagnosis of SCJ infection is in question, radio-isotope scans are highly sensitive for detection of osteomyelitis [13]. When swelling and erythaema are present, needle aspiration for both bacteriological diagnosis and therapeutic drainage may be attempted. A CT scan with fine cuts or magnetic resonance imaging scans should be obtained in infections that fail to resolve, to define the extent of spread. Diagnostic CT findings include periosteal reaction, bony sequestra, reactive sclerosis, sinus tracts and air fluid levels [13].

The majority of early SCJ infections settle with conservative measures, as long as no abscess has formed and infection has not spread to the mediastinum. On average, the duration of antibiotic required has been reported to be as high as 52 days (70 days in our patient) [14].

In cases where abscesses have developed, drainage and thorough debridement is necessary. Excision of the medial end of the clavicle, first rib and manubrium may be necessary at times. This usually leaves a large chest wall defect and major vessels uncovered. This can be rectified by an advancement flap or rotational flap of the pectoralis major muscle [7,15].

## Conclusion

SCJ infections are rare, often have an insidious onset, and require a high degree of clinical suspicion. Vague symptoms of neck and shoulder pain may cloud the initial diagnosis, as was the case in our patient. Surgical intervention is often required; however, our patient avoided major intervention and settled with parenteral antibiotics and washout of the joint.

## Abbreviations

CT: computed tomography; CVC: central venous catheter; MRSA: methicillin-resistant *Staphylococcus aureus*; SCJ: sternoclavicular joint; WBC: white blood cell count.

## Competing interests

The authors declare that they have no competing interests.

## Authors' contributions

CP and NJ drafted the case study and performed the initial literature review. NFSW, RC and JEP critically appraised and revised the manuscript. All authors have read and approved the final manuscript.

## Consent

Written informed consent was obtained from the patient for publication of this case report and any accompanying images. A copy of the written consent is available for review by the Editor-in-Chief of this journal.
